# Deep Learning Performance in Analyzing Nailfold Videocapillaroscopy Images in Systemic Sclerosis

**DOI:** 10.3390/diagnostics15222912

**Published:** 2025-11-18

**Authors:** Müçteba Enes Yayla, Ayhan Aydın, Mahmut Kılıçaslan, Mürüvvet Kalkan, Mehmet Serdar Güzel, Aida Shikhaliyeva, Serdar Sezer, Emine Uslu, Aşkın Ateş, Tahsin Murat Turgay

**Affiliations:** 1 Division of Rheumatology, Department of Internal Medicine, Faculty of Medicine, Ankara University, Ankara 06230, Turkey; ada.shikh@gmail.com (A.S.); serdarsezer1987@hotmail.com (S.S.); drusluemine@gmail.com (E.U.); askinates1970@hotmail.com (A.A.); tmturgay@hotmail.com (T.M.T.); 2 Nallihan Vocational School, Computer Technologies Department, Ankara University, Ankara 06920, Turkey; ayaydin@ankara.edu.tr; 3 Information Technologies Vocational School, Department of Statistics, Ankara University, Ankara 06100, Turkey; m.kilicaslan@ankara.edu.tr; 4 Department of Computer Engineering, Ankara University, Ankara 06100, Turkey; kalkanm@ankara.edu.tr (M.K.); mguzel@ankara.edu.tr (M.S.G.)

**Keywords:** artificial intelligence, deep learning, nailfold videocapillaroscopy, systemic sclerosis, classification

## Abstract

**Background/Objectives**: The aim of this study was to classify nailfold videocapillaroscopy (NVC) images obtained from patients with systemic sclerosis (SSc) and healthy individuals using deep learning methods. **Methods**: Between January and June 2025, 1280 NVC images were recorded from 50 SSc and 30 healthy individuals. The images were classified by two rheumatologists as normal, early, active, and late-stage SSc patterns. After removing 191 unclassifiable and 112 low-quality images, 977 usable images remained. To ensure balanced classes, 245 normal images were excluded. The final dataset was split into training (70%), validation (20%), and test (10%) sets. Six different deep learning models (MobileNetV3Large, ResNet152V2, Xception, VGG-19, InceptionV3, and NASNetLarge) with varying levels of complexity and computational efficiency were selected to compare their performance. Accuracy, precision, recall, F1 Score, and cross-entropy loss were employed as performance metrics. These metrics are commonly used in the literature to evaluate the effectiveness of classification models. **Results**: Deep learning models achieved an accuracy ranging from 90.6% to 98.9%, a precision of 93.4% to 98.9%, a recall of 90.6% to 98.8%, an F1 score of 92% to 98.9%, and an ROC AUC performance between 99% and 100%. InceptionV3 demonstrated the best performance with an accuracy of 98.95%, a precision of 98.94%, a recall of 98.80%, and an F1 score of 98.88%. In terms of test loss, the lowest values were observed in the InceptionV3 and NasNetLarge models, both with a loss of 0.03. Overall, the ROC AUC values for all models ranged between 98.99% and 100%, indicating competitive performance. **Conclusions**: The current findings suggest that deep learning methods may be capable of classifying NVC images as accurately as experienced rheumatologists.

## 1. Introduction

Systemic sclerosis (SSc) is a systemic autoimmune disease characterized by microvascular damage, autoimmunity, and fibrosis [1]. Nailfold video capillaroscopy (NVC) is a simple, inexpensive, and non-invasive method for observing microvascular changes and recommended for use in the diagnosis and classification of SSc [2,3]. Abnormal findings such as dilated or enlarged capillaries, microhemorrhages, decreased capillary density, and capillary architecture abnormalities may be observed in patients with SSc. In addition, specific capillary scoping staging such as early, active, and late patterns can be performed based on the presence and frequency of these abnormal findings in patients with systemic sclerosis [2,4,5]. There are also studies claiming that these stages may be related to the activity, severity, and prognosis of the disease [6].

Although NVC is a simple method to apply, it must be applied in accordance with certain standards [7]. There are known difficulties for rheumatologists in clinical practice. First, interpreting images requires specific training and experience. Furthermore, NVC is a relatively time-consuming procedure, as it involves evaluating four fingers on both hands of a patient. The interpretation of the images obtained is subject to subjectivity when the human factor is involved. Therefore, automation is important in the evaluation of images.

Studies have emerged that have achieved successful results in the analysis of NVC images through the use of artificial intelligence, machine learning, and deep learning in the field of medicine. In these studies, evaluations based on these techniques have achieved at least the same level of accuracy as human-based evaluations [8,9,10,11]. In this study, we aimed to classify NVC images obtained from SSc patients and healthy individuals using deep learning methods. The contribution of this study is that the proposed framework provides a comprehensive comparison of six state-of-the-art deep learning architectures on a balanced multi-class dataset representing normal, early, active, and late SSc stages. Furthermore, the use of real clinical NVC images from patients and healthy individuals, rather than synthetic or publicly available datasets, will inspire future studies.

## 2. Materials and Methods

### 2.1. Participants

Between January 2025 and June 2025, 50 patients with SSc and 30 healthy volunteers who applied to the Rheumatology Division Outpatient Clinic of the Department of Internal Medicine, Faculty of Medicine, Ankara University, were included in the study. SSc patients were selected according to the 2013 American College of Rheumatology (ACR)/European Alliance of Associations for Rheumatology (EULAR) classification criteria for SSc [3]. Those with other rheumatic diseases or diabetes mellitus were excluded from the study.

Ethical approval for the study was obtained from the Ankara University Faculty of Medicine Human Research Ethics Committee on 21 November 2024, under decision number İ10-759-24. Written informed consent was obtained from all participants.

### 2.2. Obtaining NVC Images

**Figure 1:**
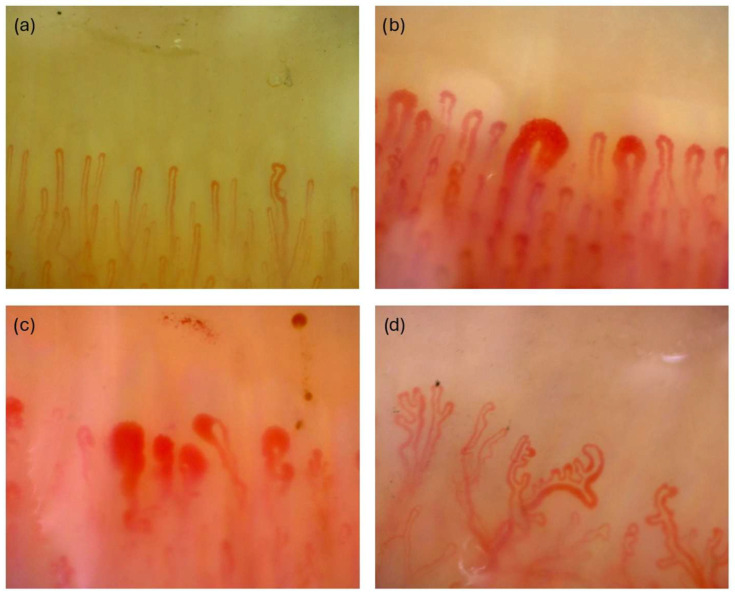
Examples of NVC image classification. (**a**) Normal NVG image, (**b**) early SSc pattern, (**c**) active SSc pattern, (**d**) late SSc pattern.

NVC images were obtained using the Dino-Lite CapillaryScope 200 Pro (Dino-Lite Europe Co., Almere, The Netherlands), a small USB handheld microscope videocapillaryscope device. The microscope was used alongside a standard Microsoft Windows laptop with DinoCapture software, version 2.0. The images were 1.80 mm × 1.3 mm and 1280 × 1024 pixels. Two images were taken from each participant’s right and left hands, from the 2nd, 3rd, 4th, and 5th fingers, at 11 o’clock and 1 o’clock positions. A total of 1280 images were obtained from 50 systemic sclerosis patients (800 images) and 30 healthy controls (480 images). The images were then classified separately as normal, early, active, or late SSc patterns by two rheumatologists experienced in capillaroscopy (MEY, EU) (Figure 1). When rheumatologists classified the images, if they did not reach the same conclusion in classifying the image, those images were classified as unclassifiable. When inter-rater reliability between the two observers was evaluated, a κ value of 0.766 (*p* < 0.001) was obtained, indicating a substantial agreement between the observers. A total of 191 images were deemed unclassifiable, and 112 images were excluded due to poor image quality. Out of the remaining 977 images, to maintain a balanced class distribution, 245 images—half of the 490 images in the normal class—were excluded from classification. The dataset was then divided into training, validation, and test sets following a 70%, 20%, and 10% split, respectively. The distribution of images by class and dataset split is presented in Table 1.

### 2.3. Data Preparation

Before loading and processing the images, several important settings are configured. These settings include the batch size (32), image size (128 × 128), and a constant seed value for all random operations. The batch and image sizes are chosen based on common standards in deep learning. The seed ensures the reproducibility of the experiments and is consistently applied to all random processes, including shuffling, augmentation, and data splitting.

After these configurations, data preprocessing is performed. This step involves resizing images, shuffling data, applying augmentations, and rescaling pixel values. During augmentation, each image is randomly flipped, rotated, and contrast-adjusted to enhance the model’s robustness and generalization. Finally, pixel values composed of RGB components are rescaled from [0, 255] to either [−1, 1] or [0, 1], depending on the model’s input requirements. Finally, the dataset is then randomly divided into training, validation, and testing sets with proportions of 70%, 20%, and 10%, respectively.

### 2.4. Deep Learning Approach

Deep learning is widely preferred in medical image analysis due to its ability to automatically extract features, achieve high accuracy, and provide interpretable results. In our work, six different models with varying levels of complexity and computational efficiency were selected to compare their performance. The selected models are MobileNetV3Large, ResNet152V2, Xception, Visual geometry group (VGG)-19, InceptionV3, and NASNetLarge. MobileNetV3Large is a convolutional neural network optimized to run on mobile and embedded systems, designed to be computationally efficient. It provides channel-wise attention mechanism through depthwise separable convolutions and Squeeze-and-Excitation (SE) blocks. Instead of the rectified linear unit (ReLU), it uses the Hardswish activation function, and its design has been automatically optimized using Neural Architecture Search (NAS) [12]. The model contains approximately 5.4 million parameters, allowing it to run quickly and with low latency on mobile devices; however, its accuracy may be limited compared to deeper models when working with very large datasets. ResNet152V2 is a 152-layer architecture developed to address the vanishing gradient problem encountered during the training of very deep neural networks. Thanks to residual connections, the gradient flow is facilitated, making the training process more stable. The V2 version improves performance by adopting an ordering of batch normalization first, then activation, and finally the weight layer [13]. The model contains approximately 60 million parameters and provides high accuracy; however, due to its high computational and memory requirements, it is not suitable for mobile applications The Xception architecture is designed as an extended version of the Inception architecture to fully exploit the potential of depthwise separable convolutions. In this model, a convolution is applied separately on each channel, which is then combined using a 1 × 1 convolution (pointwise convolution). With approximately 22 million parameters, Xception offers strong parameter efficiency, but it can be sensitive to optimization parameters during training [14]. The VGG-19 model aims to provide a deep neural network architecture with a simple and regular structure. The model consists of a total of 19 layers (16 convolutional + 3 fully connected) using only 3 × 3 convolution kernels and 2 × 2 max pooling. With approximately 143 million parameters, this architecture requires high computational power and memory, and has fallen behind more efficient modern architectures for practical applications [15]. InceptionV3 is designed to combine wide and deep network structures without incurring high computational costs. Inception blocks apply convolution kernels of different sizes (1 × 1, 3 × 3, 5 × 5) simultaneously, and the outputs are merged. Using factorized convolution techniques (e.g., replacing a 5 × 5 convolution with two consecutive 3 × 3 convolutions), computational cost is reduced. The model has approximately 24 million parameters and provides high accuracy with efficiency [16]. The NASNetLarge model has an automatically optimized architecture using Neural Architecture Search (NAS). At its core, it consists of repeated normal and reduction cells, providing high accuracy with approximately 88 million parameters. However, due to its large size and high computational requirements, it can only run effectively on powerful hardware [17].

**Figure 2:**
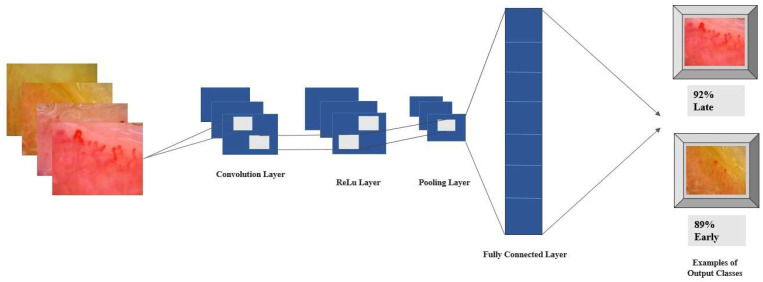
Architecture of the proposed deep learning model for classification.

All models were trained and evaluated using transfer learning with ImageNet pre-trained weights to achieve high performance with limited data and reduced training time. The first one-third of layers were frozen to retain ImageNet features, while the rest were fine-tuned for domain adaptation. Training lasted 50 epochs with the Adam optimizer (learning rate 1 × 10^−4^). A dropout rate of 0.2 was applied during each forward pass to reduce overfitting. To ensure reliable and reproducible results, each model was trained and evaluated five times under identical conditions, and the final metrics represent the average performance across runs (Figure 2).

### 2.5. Model Evaluation Metrics

The models’ performance was evaluated and compared using standard metrics, including accuracy, cross-entropy loss, precision, recall (sensitivity), F1-score, and Receiver-operating characteristic curve (ROC) area under the curve (AUC), providing insights into the advantages of different deep learning architectures in predicting disease stages from capillaroscopy images of nailfold capillaries.

The number of correct predictions made by a model is what is meant by ‘accuracy’. Accuracy takes a value between 0 and 1, and the closer it is to 1, the stronger the model’s ability to make correct predictions. Precision is the ratio of true positives to the number of samples predicted as positive by the model. Precision takes a value between 0 and 1. The model is more likely to avoid misclassifying negative samples as positive if the value is closer to 1. The proportion of true positive samples that the model correctly predicted is known as the recall (sensitivity). Recall takes a value between 0 and 1. Its proximity to 1 indicates that the model is suitable for predicting positive instances. The F1 score is the harmonic mean of precision and recall and valued from 0 to 1. A value close to 1 indicates that the model has a better balance between precision and recall. The calculation of measurement parameters is presented in Table 2. ROC is a probability curve, and the area under it, the AUC, represents the degree or measure of separability. The ability to distinguish between classes improves as the AUC increases. Cross-entropy loss measures the difference between the model’s prediction probability distribution and the labels.

## 3. Results

Among a total of 50 patients with SSc, 92% were female (46/50), 14% (7/50) had diffuse cutaneous SSc, 72% (36/50) had limited cutaneous SSc, and 14% (7/50) had sine scleroderma. The mean age of the patients was 58.7 ± 11.1 years, and the median disease duration was 5.6 years [interquartile range: 16.3]. Among the SSc patients, 98% were positive for antinuclear antibodies, 24% for anti-topoisomerase I, 64% for anti-centromere, and 12% for anti-PM/Scl antibodies.

The program was executed in Google Colab using the hardware of a TPU v2 (8 cores, 64 GB HBM, 700 MHz) and the software stack of Python 3.10, TensorFlow 2.19.0, and NumPy 2.0.2.

**Figure 3:**
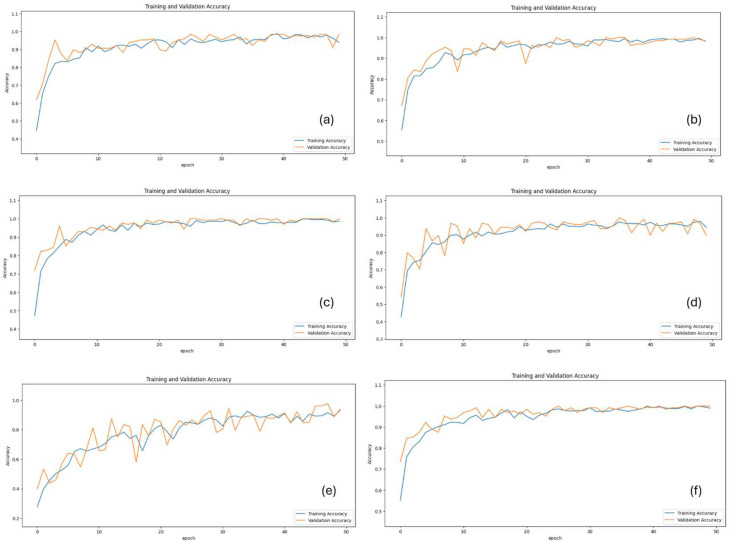
Training and validation accuracy over epochs. (**a**) Inception V3; (**b**) MobileNetV3Large; (**c**) NasNetLarge; (**d**) ResNet152V2; (**e**) VGG-19; (**f**) Xception.

**Figure 4:**
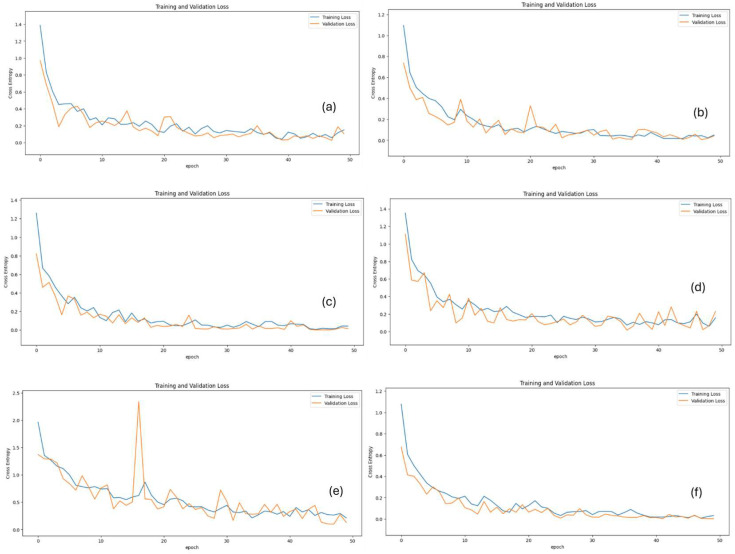
Training and validation loss over epochs. (**a**) Inception V3; (**b**) MobileNetV3Large; (**c**) NasNetLarge; (**d**) ResNet152V2; (**e**) VGG-19; (**f**) Xception.

**Figure 5:**
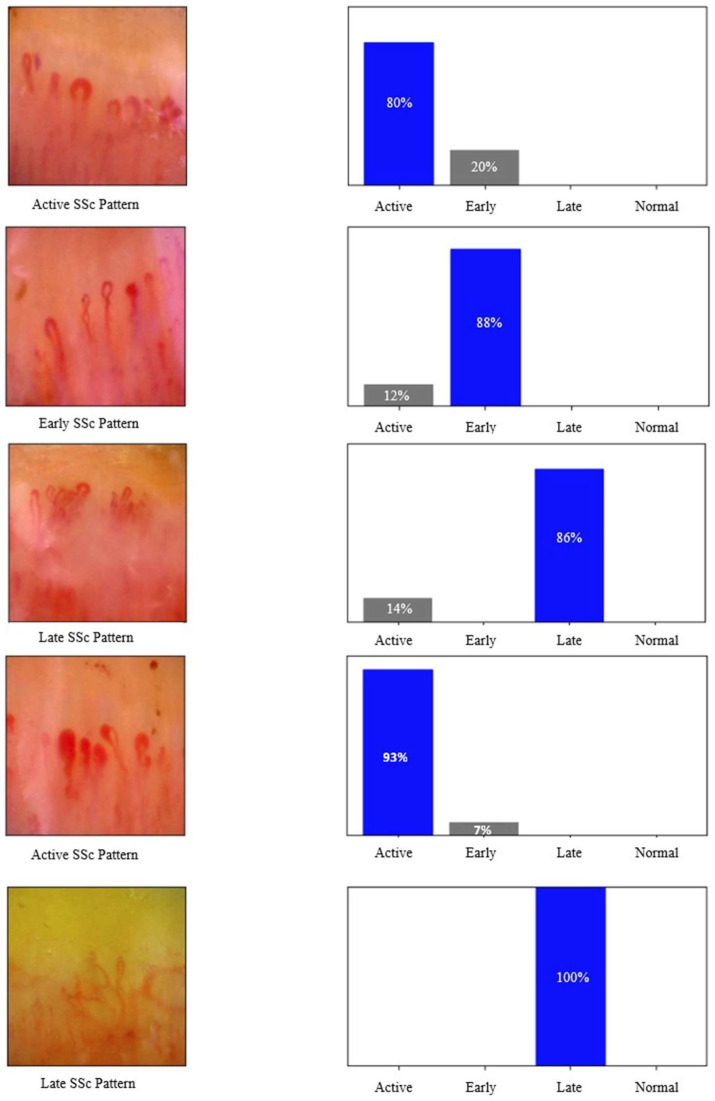
Test samples classified by deep learning methods.

Model performances were evaluated using metrics such as test loss, accuracy, precision, recall, F1 score, and ROC AUC. The MobileNetV3Large model achieved 95.83% accuracy and 100% ROC AUC, demonstrating high performance. The MobileNetV3Large model also presented a strong balance with 96.87% precision and 94.49% recall. The Xception model also yielded highly successful results with 96.87% accuracy, a 96.95% F1 score, and 99.95% ROC AUC. The ResNet152V2 model showed the lowest performance, with an accuracy of 90.62% and an F1 score of 91.98%. The VGG-19 model provided balanced performance, achieving 94.79% accuracy and 98.99% ROC AUC. The InceptionV3 model reached the highest performance, with 98.95% accuracy, a 98.88% F1 score, and 99.99% ROC AUC. The InceptionV3 model also achieved 98.94% precision and 98.80% recall, minimizing both false positives and false negatives. Similarly, the NasNetLarge model delivered impressive results with 97.91% accuracy, a 98.06% F1 score, and 100% ROC AUC. In terms of test loss, the lowest values were observed in the InceptionV3 and NasNetLarge models, both with a loss of 0.03. Overall, the ROC AUC values for all models ranged between 98.99% and 100%, demonstrating that each architecture effectively distinguished among the four SSc stages (normal, early, active, and late) with competitive classification performance. The performance of the methods with various metrics is represented in Table 3 and Table 4 and Figure 3 and Figure 4. Per-class accuracy results are shown in Table 5. Sample images of the results obtained from deep learning methods on test samples are presented in Figure 5.

## 4. Discussion

The present study assessed the performance of six distinct deep learning models in classifying NVC images into normal, early, active, and late SSc patterns. All models demonstrated highly satisfactory performance metrics. These findings suggest that deep learning methods can successfully classify NVC images without the need for human input. The evaluation of NVC images, considering the busy outpatient clinic settings, can increase the workload of clinicians. The application of deep learning approaches may facilitate the diagnostic process and significantly reduce the clinicians’ workload.

Automation systems, machine learning, and deep learning methods have been investigated in previous studies for the classification of NVC images. For the first time in 2012, Doshi et al. examined the evaluation of NVC images in SSc patients using artificial intelligence and achieved a prediction correction of 70–75% [18]. Subsequently, studies emerged on the evaluation of automation systems in the assessment of NVC images using artificial intelligence [8,9]. Berks et al. aimed to assess capillary morphology using a layered machine learning approach and reported that the automation systems achieved an accuracy of 93.6% and a precision of 64.1% in detecting distal capillaries [8]. Cutolo et al. aimed to determine capillary density and count using an automation system. The software they developed, named AUTOCAPI, demonstrated an 88–94% correlation with the counting of capillaries by experienced rheumatologists in SSc patients, 62–85% in patients with primary Raynaud’s phenomenon, and 56–91% in healthy individuals [9]. The study, which used two deep learning methods, U-Nets and ResNet34, showed that artificial intelligence was as successful as SSc specialists in distinguishing NVC images in high- and low-resolution images [11]. In a study investigating the performance of a vision transformer (ViT)-based deep learning model and an off-the-shelf AI solution in identifying microangiopathy-specific features in the NVC images of SSc patients from the European Scleroderma Trials and Research group (EUSTAR) and the Very Early Diagnosis of Systemic Sclerosis (VEDOSS) cohorts, the ViT demonstrated quite good performance in detecting microangiopathic changes (AUC 81.8–84.5%). In addition, the ViT’s ability to accurately identify early, active, or late scleroderma patterns ranged from 85.8% to 93.5% [10]. Jalal et al. evaluated the performance of a model developed to distinguish between normal and abnormal NVC images using a cascade transfer learning approach based on EfficientNet-B0. By transferring learning domain-specific classes to an EfficientNet-B0 model pre-trained on the ImageNet dataset, they achieved 100% accuracy, precision, recall, F1 score, and ROC AUC values [19]. In a study comparing the performance of machine learning and deep learning methods using images from the SCLEROCAP study, it was shown that three deep learning models—ResNet18, VGG-16, and DenseNet-121—outperformed the machine learning approaches. In this study, the performance metrics of the deep learning methods were reported as follows: accuracy ranged from 87% to 94%, F1 score ranged from 85% to 94%, and AUC ranged from 87% to 95% [20]. Similarly, in our study, we also demonstrated that deep learning methods performed well, as shown in the aforementioned studies. In our analysis evaluating six deep learning models, we found that our performance metrics were as follows: accuracy ranged from 91% to 99%, precision ranged from 93% to 99%, recall ranged from 91% to 99%, F1 score ranged from 92% to 99%, and ROC AUC ranged from 99% to 100%.

Among the evaluated models, InceptionV3 achieved the highest overall accuracy and the most consistent per-class performance, likely due to its multi-scale feature extraction and effective use of inception modules that capture both fine and coarse spatial details. NasNetLarge ranked second in overall performance, benefiting from its architecture search-optimized design that enhances feature diversity and generalization across classes. Xception followed closely, demonstrating competitive accuracy and balanced per-class outcomes through efficient spatial–channel decoupling enabled by depth-wise separable convolutions. In contrast, ResNet152V2 exhibited the weakest overall and per-class performance, suggesting a mild overfitting tendency or suboptimal adaptation to the dataset’s scale and characteristics. The relatively balanced class distribution supports these outcomes, indicating that the superior results of the top models stem primarily from architectural strengths rather than data bias. However, minor per-class variations, particularly in the early and late categories, indicate limited generalization in finer stage differentiation, likely influenced by the small dataset size. Across repeated training runs, low standard deviations confirmed that model performance was stable and not overly sensitive to random initialization.

The present study has several limitations that should be acknowledged. The exclusion of images that could not be consistently classified by two rheumatologists may have introduced selection bias. Furthermore, the dataset was derived from a single institution using a single imaging device, which inherently limits sample diversity and increases the potential risk of overfitting. Although the results were highly consistent across repeated experiments, the relatively small, single-center dataset may restrict the generalizability of the findings to broader populations or different imaging conditions. Therefore, future research should include external validation using larger, multi-center datasets to confirm the robustness, reproducibility, and clinical applicability of the proposed deep learning approach.

## 5. Conclusions

The current findings prove that deep-learning-based systems can classify NVC images with accuracy levels up to 98.9%, comparable to the diagnostic performance of experienced rheumatologists. These results support the integration of deep learning algorithms into clinical workflows for objective and consistent assessment of microvascular changes in SSc. The automatic staging of capillaroscopy images holds significant potential for clinical decision support systems. In the future, as more consistent and accurate artificial-intelligence-based systems are developed, these systems could automatically report NVC images in clinics with high patient volume and contribute to rheumatology education through training programs focused on NVC image interpretation. Furthermore, AI-assisted web- or mobile-based applications could be developed to support clinicians with limited experience in NVC interpretation by enabling remote evaluation of NVC images.

In summary, deep learning models, particularly the InceptionV3 architecture, achieved high accuracy in the automated classification of NVC images in SSc. These findings highlight the potential of artificial-intelligence-based approaches to assist clinicians in objective and consistent image interpretation. However, the current results should be confirmed in larger, multi-center datasets and through prospective clinical validation studies before routine clinical implementation. Future research is planned to enhance the robustness and clinical applicability of the proposed approach. Model explainability techniques such as Grad-CAM will be integrated to visualize key image regions influencing predictions. The dataset will be expanded with larger, multi-center cohorts from diverse imaging devices, and non-deterministic factors will be controlled to ensure reproducibility. Finally, integration of the developed models into diagnostic workflows and their evaluation in prospective clinical settings are planned to assess their practical value in SSc assessment.

## Figures and Tables

**Table 1:** Distribution snapshot of dataset splits.

Class	Training	Validation	Test	Total
Active	122	35	17	174
Early	115	33	16	164
Late	104	30	15	149
Normal	172	49	24	245
Total	513	147	72	732

**Table 2:** Calculation of evaluation metrics.

Accuracy	$\frac{TP+TN}{TP+TN+FP+FN}$
Precision	$\frac{TP}{TP+FP}$
Recall	$\frac{TP}{TP+FN}$
F1 Score	$\frac{2\times TP}{2\times TP+FP+FN}$
Cross-entropy loss	$L=-\frac{1}{m}\sum_{i=1}^{m}Yi\cdot \log\left(p\right)$

FP, false positive; FN, false negative; TP, true positive; TN, true negative.

**Table 3:** Evaluation of deep learning model performance by loss, accuracy, and ROC AUC.

Model	Loss	Accuracy	ROC AUC
MobileNetV3Large	0.08 ± 0.01	95.83% ± 0.20%	100.00% ± 0.00%
ResNet152V2	0.26 ± 0.04	90.62% ± 0.23%	99.98% ± 0.06%
Xception	0.07 ± 0.01	96.87% ± 0.08%	99.95% ± 0.01%
VGG-19	0.18 ± 0.02	94.79% ± 0.11%	98.99% ± 0.10%
InceptionV3	0.03 ± 0.01	98.95% ± 0.08%	99.99% ± 0.01%
NasNetLarge	0.03 ± 0.01	97.91% ± 0.08%	100.00% ± 0.00%

**Table 4:** Evaluation of deep learning model performance by precision, recall, and F1-Score.

Model	Precision	Recall	F1 Score
MobileNetV3Large	96.87% ± 0.19%	94.49% ± 0.25%	95.66% ± 0.20%
ResNet152V2	93.38% ± 0.27%	90.62% ± 0.29%	91.98% ± 0.26%
Xception	96.83% ± 0.10%	97.08% ± 0.09%	96.95% ± 0.2%
VGG-19	95.52% ± 0.12%	91.60% ± 0.19%	93.52% ± 0.19%
InceptionV3	98.94% ± 0.02%	98.80% ± 0.05%	98.88% ± 0.01%
NasNetLarge	98.21% ± 0.08%	97.91% ± 0.09%	98.06% ± 0.09%

**Table 5:** Evaluation of deep learning model performance by accuracy per class.

Model	Normal	Active	Early	Late
MobileNet V3 Large	96.12% ± 0.31%	95.40% ± 0.44%	94.82% ± 0.3%	97.5% ± 0.2%
ResNet152 V2	91.34% ± 0.37%	90.22% ± 0.31%	89.60% ± 0.54%	91.80% ± 0.41%
Xception	97.03% ± 0.16%	96.80% ± 0.31%	96.33 ± 0.29%	97.94 ± 0.21%
VGG-19	94.56% ± 0.30%	93.81% ± 0.34%	92.70 ± 0.39%	95.93 ± 0.%
Inception V3	99.04% ± 0.12%	98.82% ± 0.19%	98.60 ± 0.12%	99.21 ± 0.14%
NasNet Large	98.25% ± 0.23%	97.90% ± 0.20%	97.40 ± 0.31%	98.70 ± 0.24%

## Data Availability

Data available on request due to restrictions (privacy and ethical reasons).

## Abbreviations

The following abbreviations are used in this manuscript:

ACR	American College of Rheumatology
AUC	Area under curve
EULAR	European Alliance of Associations for Rheumatology
EUSTAR	European Scleroderma Trials and Research
NAS	Neural architecture search
NVC	Nailfold videocapillaroscopy
ReLU	Rectified linear unit
ROC	Receiver-operating characteristic curve
SE	Squeeze-and-Excitation
SSc	Systemic sclerosis
VEDOSS	Very Early Diagnosis of Systemic Sclerosis
VGG	Visual geometry group
ViT	Vision transformer
